# Can Indoor Swimming Alter Hormones in Boys?

**DOI:** 10.1289/ehp.120-a18

**Published:** 2012-01-01

**Authors:** Bob Weinhold

**Affiliations:** Bob Weinhold, MA, has covered environmental health issues for numerous outlets since 1996. He is a member of the Society of Environmental Journalists.

Swimming is generally considered an excellent form of exercise, and indoor swimming is common, especially in winter. However, there is evidence that swimming in a chlorinated indoor pool can cause respiratory irritation or genotoxicity in some people.^1^ A recent study suggests another possible consequence: altered levels of hormones in boys.^2^

The study population consisted of 199 primarily white boys aged 14-18 years who swam regularly in indoor and/or outdoor chlorinated pools, and 162 similar boys who swam most frequently in an indoor pool disinfected with copper–silver ionization (but also swam at times in indoor or outdoor chlorinated pools). The authors compared serum levels of several testicular hormone biomarkers between the two groups: inhibin B, total and free testosterone, sex hormone–binding globulin, luteinizing hormone, follicle-stimulating hormone, and dehydroepiandrosterone sulphate.

The boys who swam the most in indoor chlorinated pools had concentrations of inhibin B and total testosterone about 20% lower than those of boys who swam in the pool disinfected with copper–silver ionization, and the former were about 3 times more likely than the latter to have abnormally low concentrations of these hormones. The effects were more pronounced for exposure before age 7 than before age 10 (after which no significant changes were seen), and adverse effects were associated with swimming as little as 30 minutes every 2 weeks.

**Figure:**
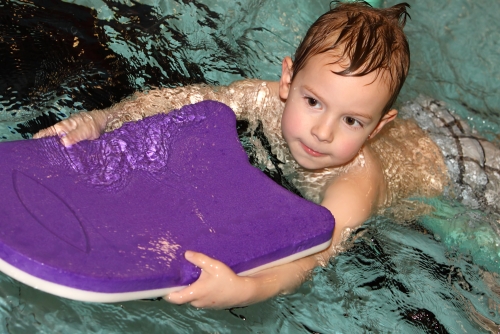
Having swum in an indoor chlorinated pool before age 7 was associated with the greatest hormonal changes in teenage boys. Andre Bonn/Shutterstock.com

There were no significant hormonal changes in boys who swam in outdoor chlorinated pools. These were primarily backyard pools that study coauthor Alfred Bernard, a professor of toxicology at Catholic University of Louvain, says tend to be less prone than public pools to have elevated concentrations of urine and other organic matter. That means less chlorination by-products are formed.

Bernard and coauthor Marc Nickmilder, also of Catholic University of Louvain, accounted for factors such as age, body mass index, time of day of blood sampling, smoking status, having been breastfed, consumption of tap or bottled water since babyhood, insecticide use, residential proximity to a busy road, and participation in certain other sports. There are several limitations to the study, such as the lack of many types of measurements of water quality and disinfection by-products in the pools and the absence of data on testis size and other indicators of each boy’s pubertal status. The clinical significance of the hormonal changes identified is therefore unclear.

The authors speculate the hormonal changes may have occurred because of exposure to chlorination by-products that permeate the scrotum and affect the testes. The authors explain that the skin of the scrotum is quite permeable for some substances and may be especially so in the warm, wet conditions of a pool. They can’t definitively say that the hormone reductions observed will cause reproductive harm, but they conclude that the potential for reproductive problems such as reduced sperm production is plausible.

The study seems well conducted, despite its limitations, says Mark Nieuwenhuijsen, a research professor at the Centre for Research in Environmental Epidemiology (CREAL). But he is surprised that the adverse impacts are limited to just inhibin B and total testosterone, because luteinizing hormone and follicle-stimulating hormone are typically considered to be involved in similar pathways. He says it is important to remember, in evaluating these findings, that epidemiological studies on disinfection by-products and semen quality have shown little or no effects, and that swimming offers significant health benefits through physical activity.

Shanna Swan, a professor of preventive medicine at Mt. Sinai School of Medicine, finds the study intriguing but unconvincing due to factors such as the different hormone effects after swimming in indoor and outdoor pools, which tend to have roughly similar chlorination treatment. She also pointed to the paucity of evidence from other studies supporting the idea that the doses the boys received could do such damage,^3^ and she says effects from bath water exposures should have been considered. Bernard says those data weren’t available and that bath water likely is different from pool water due to the presence of fewer organics such as urine. But he agrees the variable is important and says it is something he plans to test in the future.

Cristina Villanueva, an investigator at CREAL, thinks the study’s findings seem plausible in the context of limited evidence from other studies. But she also remains wary: “This hypothesis has barely been evaluated in humans. Consequently, given that this is the first epidemiological study on the topic, interpretation should be cautious until confirmed in new studies.”

Further research would be helpful, says David Savitz, a professor of epidemiology at Brown University: “While there is no suggestion [in this study] of changes that result in severe deficiencies, on a population level there is a range of fertility potential, and any influence that reduces the capability for the entire exposed population will cause clinically identifiable problems in a subset.”
